# Supplementary material for: Empirical methods for controlling false positives and estimating confidence in ChIP-Seq peaks
Source: BMC Bioinformatics. 2008 Dec 5;9:523. doi: 10.1186/1471-2105-9-523 (PMC2628906; doi:10.1186/1471-2105-9-523)
Supplement: Additional File 1 — A variety of html documents from the USeq web site detailing the available applications, their best usage, output file type descriptions, command line menus, etc. [file 1471-2105-9-523-S1.zip › USeqUserGuides/JavaDoc.zip!/JavaDoc/edu/utah/seq/data/class-use/EnrichedRegion.html]

Uses of Class edu.utah.seq.data.EnrichedRegion


|  |  |  |
| --- | --- | --- |
| **Overview** \| **Package** \| **Class** \| **Use** \| **Tree** \| **Deprecated** \| **Index** \| **Help** | |  |
| PREV   NEXT | **FRAMES**    **NO FRAMES**     **All Classes** |  |


---


## **Uses of Class edu.utah.seq.data.EnrichedRegion**

| Packages that use EnrichedRegion | |
| --- | --- |
| **edu.utah.seq.analysis** | Primary analysis applications and helper classes for working with binary PointData, xxx.bar.zip files. |

Uses of EnrichedRegion in edu.utah.seq.analysis

| Methods in edu.utah.seq.analysis with parameters of type EnrichedRegion | |
| --- | --- |
| `static void` | `EnrichedRegionMaker.printEGRFile(EnrichedRegion[] ers, java.io.File egr, java.lang.String genomeVersion, java.lang.String[] scoreLabels)`             Writes out an egr file, used by IGB for graph display. |
| `static void` | `EnrichedRegionMaker.printEGRFileForBestSubWindow(EnrichedRegion[] ers, java.io.File egr, java.lang.String genomeVersion)`             Writes out an egr file for best sub windows, used by IGB for graph display. |
| `static void` | `EnrichedRegionMaker.printEnrichedRegionReport(EnrichedRegion[] ers, java.io.File spreadSheetFile, java.lang.String genomeVersion, java.lang.String[] scoreDescriptions)`             Writes out an excel compatible tab delimited spreadsheet with a hyperlink to IGB. |
| `static void` | `EnrichedRegionMaker.printGFFFile(EnrichedRegion[] ers, java.io.File egr, java.lang.String genomeVersion, int index, java.lang.String[] scoreLabels)`             Prints a simple gff file, the index is used to pick a score to place in the score column. |
| `static void` | `EnrichedRegionMaker.printGFFFileForBestSubWindow(EnrichedRegion[] ers, java.io.File egr, java.lang.String genomeVersion)`             Prints a simple gff file, the index is used to pick a score to place in the score column. |
| `void` | `EnrichedRegionMaker.printSubWindowGraphFiles(EnrichedRegion[] ers, java.io.File saveDir, java.lang.String genomeVersion)` |

Constructors in edu.utah.seq.analysis with parameters of type EnrichedRegion

`PeakPicker(EnrichedRegion[] enrichedRegions,
java.io.File[] treatmentPointDirs,
java.io.File[] controlPointDirs,
int windowSize)`
  
          Picks the best peak based on rescanning each enriched region using the defined window size.

---


|  |  |  |
| --- | --- | --- |
| **Overview** \| **Package** \| **Class** \| **Use** \| **Tree** \| **Deprecated** \| **Index** \| **Help** | |  |
| PREV   NEXT | **FRAMES**    **NO FRAMES**     **All Classes** |  |


---
